# *IFITM3* variants point to a critical role in emergent virus infections

**DOI:** 10.1128/mbio.03347-24

**Published:** 2025-04-16

**Authors:** Parker J. Denz, Jacob S. Yount

**Affiliations:** 1 Department of Microbial Infection and Immunity, The Ohio State University College of Medicine 683676 , Columbus, Ohio, USA; 2 Viruses and Emerging Pathogens Program, Infectious Diseases Institute, The Ohio State University 2647, Columbus, Ohio, USA; Albert Einstein College of Medicine, Bronx, New York, USA

**Keywords:** IFITM3, influenza virus, SARS-CoV-2, innate immunity, SNP, emerging viruses

## Abstract

Interferon-induced transmembrane protein 3 (IFITM3) is a cellular protein that restricts numerous viral infections by blocking virus–host membrane fusion. In humans, there are two *IFITM3* single nucleotide polymorphisms (SNPs), rs12252-C and rs34481144-A, that decrease IFITM3 activity and have been associated with severe illness following influenza virus infections. Mice lacking IFITM3 show increased influenza severity, supporting this association. However, some studies do not find a consistent link between *IFITM3* SNPs and infection severity, causing uncertainty about its role *in vivo*. Review of the literature indicates that *IFITM3* SNPs are primarily associated with increased viral disease in infections with emergent influenza viruses, such as the 2009 H1N1 pandemic virus and zoonotic H7N9 virus. Similarly, *IFITM3* SNPs are reported to be risk factors for increased severity in other emergent infections, including SARS-CoV-2, Hantaan virus, and HIV. In contrast, most studies that failed to find an association examined seasonal influenza. We posit that adaptive immune mechanisms, including pre-existing antibodies and memory T cells against seasonally circulating viruses, compensate for IFITM3 deficiencies, therefore masking its role in seasonal influenza. We propose that IFITM3 is most critical in defending against emergent viruses and should be a key focus of public health strategies to prevent the emergence and spread of novel pathogens, with individuals carrying *IFITM3* SNPs potentially benefiting from broadened vaccine coverage, avoidance of animal reservoirs, or enhanced masking to protect themselves and the wider population.

## ANTIVIRAL ACTIVITY OF IFITM3

The interferon-induced transmembrane protein (IFITM) family includes five IFITMs in humans (IFITM1, 2, 3, 5, and 10). Each of these displays antiviral activity when overexpressed, but only IFITMs 1, 2, and 3 are induced by interferons and are thought to be part of the innate antiviral immune response (1–11). Among these, IFITM3 has been studied the most extensively. The antiviral properties of IFITM3 were uncovered through siRNA screening and by overexpression studies investigating the protein as a candidate antiviral restriction factor (12–15). Early work on IFITM3 showed that it effectively inhibits infection by viruses that enter cells via endocytosis, such as influenza virus and Dengue virus, but does not inhibit murine leukemia virus or Sendai virus that fuse with the plasma membrane (12, 13). Indeed, IFITM3 possesses an endosomal sorting signal directing the protein to endosomes and lysosomes (16–20) and it has now been shown to inhibit a diverse array of viruses that rely on endocytosis for cellular entry (1, 21–23).

IFITM3 functions by modifying membrane dynamics to impede the fusion between viral and host membranes, thus preventing deposition of viral genomic material into the cytoplasm and subsequent replication. In single virus particle imaging experiments, it was revealed that hemifusion between virus and endosome membranes still occurs in cells expressing IFITM3, but fusion is stalled before the formation of a fusion pore, thus trapping viral genetic material in endolysosomes (24). This antiviral activity is mediated largely by an amphipathic helix domain that inserts into the outer leaflet of the endosomal membrane and reorganizes local cholesterol and lipid compositions to change membrane fluidity and the ability of the membranes to undergo curvature changes such that viral fusion is disfavored (6, 24–32). Post-translational modifications, including phosphorylation and palmitoylation, regulate IFITM3 localization and antiviral activity, fine-tuning its ability to restrict viral infection (7, 17–19, 33). Likewise, baseline IFITM3 levels in cells are regulated by NEDD4-mediated ubiquitination, resulting in degradation of IFITM3 in lysosomes (29, 34). During virus-induced interferon responses, NEDD4 is inhibited and IFITM3 accumulates (35, 36). Overall, IFITM3 is an endosomal membrane-associated antiviral protein subject to complex intracellular regulation.

## *IFITM3* SINGLE NUCLEOTIDE POLYMORPHISMS IN THE HUMAN POPULATION

Within the human population, there are two single nucleotide polymorphisms (SNPs) in the *IFITM3* gene that have been credibly associated with increased severity of influenza. The rs12252-C SNP is common in eastern Asian populations such that 20% of Chinese individuals are homozygous for the SNP (37). The rs12252-C SNP was proposed to introduce a splice acceptor site resulting in truncated IFITM3 missing its N-terminal 21 amino acids (38). Overexpression of truncated IFITM3 resulted in reduced ability to inhibit influenza virus infection compared to WT IFITM3 (17–20, 38). Subsequent studies observed that the truncation led to mislocalization of the IFITM3 protein due to the loss of its endosomal sorting signal (16–20). Supporting the biological plausibility of splicing alterations, diversification of IFITM family genes in primates often involves loss or mutation of the N-terminus (18, 19). Likewise, alternative IFITM RNA splicing has been reported to occur in bats and other mammals (39). However, RNA sequencing failed to identify alternatively spliced transcripts in human PBMCs from patients homozygous for the C allele (38, 40, 41), suggesting that our understanding of how this SNP alters the function of *IFITM3* remains unclear. It should be a priority of the field to determine whether lung epithelial cells, the primary targets of influenza virus infection, produce the predicted alternatively spliced *IFITM3* transcript variant when the C allele is present.

The second *IFITM3* SNP, rs34481144-A, is located within the promoter of the *IFITM3* gene and decreases mRNA and protein levels (38, 42). The rs34481144-A SNP is common in European populations, with 4% of European individuals being homozygous for the SNP (42). The mechanism by which this SNP impacts gene expression was shown to be differential occupancy of the *IFITM3* promoter by activating versus inhibitory transcription factors (42). Specifically, rs34481144-A introduces a CTCF binding site that impairs binding by interferon regulatory factors that would otherwise activate *IFITM3* transcription.

Studies in IFITM3-deficient mice further support an essential role of IFITM3 in restricting viral infection and reducing disease severity. *Ifitm3^−/−^* mice infected with influenza A virus experience increased viral loads, weight loss, lung and heart damage, and mortality when compared with WT mice (11, 38, 43, 44). Other viruses, including West Nile virus, Venezuelan equine encephalitis virus, cytomegalovirus, and SARS-CoV-2, also trigger more severe disease in *Ifitm3^−/−^* versus WT mice (45–48).

While IFITM3 is one of the factors that is most reproducibly associated with severe viral infection, other interferon-related protein defects have been associated with more severe viral infection, including TLR3, IRF3, IRF7, and OAS1 (49, 50). For example, the rs5743313 SNP found in TLR3 has been associated with more severe influenza A virus infection, perhaps through effects on interferon induction (49, 51, 52). Another study showed that SNPs in IRF3 and TLR3 are associated with more severe COVID-19, likely through a decrease in type III interferon expression (53). However, TLR3, IRF3, and IRF7 mutations are exceedingly rare. On the other hand, a more commonly observed polymorphism in OAS1 generates a protein that is less active against SARS-CoV-2 and has been associated with increased COVID-19 severity (54–56). These studies highlight how defects in other innate immunity factors may also lower barriers to viral infection in humans similar to the *IFITM3* SNPs.

## CONFLICTING REPORTS LINKING SEVERE VIRUS INFECTIONS TO HUMAN *IFITM3* SNPs

Numerous studies have shown the relationship between *IFITM3* SNPs and severe influenza, but several fail to show consistent associations with severe outcomes (37, 38, 41, 42, 51, 57–64). A summary of all published studies on *IFITM3* SNPs is shown in Table 1. We propose that the discrepancy in results arises from focusing on either emergent or seasonal viruses in the distinct studies. Emergent viruses, like the 2009 H1N1 pandemic virus, often enter populations with little or no pre-existing immunity, making the innate immune response, including IFITM3, essential for early viral control. Conversely, pre-existing immunity from past infections or vaccination against seasonal influenza allows adaptive immune responses, such as cytotoxic T cells and antibodies, to dominate, effectively compensating for IFITM3 deficiencies (65–67). The quick action of neutralizing antibodies or memory T cells may render IFITM3 non-essential in the early control of infection, thus explaining the lack of consistent associations between *IFITM3* SNPs and severe outcomes in seasonal influenza (41, 59, 60, 68).

**TABLE 1:** Summary of studies investigating the association between human *IFITM3* SNPs and disease severity^*a*^

Virus	Reference	*IFITM3* SNP	Illness severity	Disease association?
Pandemic 2009 H1N1 influenza virus	(57)	rs12252-C	Severe	Yes
	(51)	rs12252-C	Death	Yes
	(38)	rs12252-C	Severe	Yes
	(63)^*b*^	rs12252-C	Hospitalization	No
	(62)^*b*^	rs12252-C	Severe	No
H7N9 avian influenza virus	(64)	rs12252-C	Hospitalization	Yes
	(51)	rs12252-C	Death	Yes
Seasonal influenza virus	(37)	rs12252-C	Severe	Yes
	(42)^*c*^	rs34481144-A	Severe	Yes
	(41)^*c*^	rs34481144-A	Mild/severe	Yes
		rs12252-C	Severe	No
	(59)^*d*^	rs12252-C	Severe	No
	(60)	rs12252-C	Hospitalization	No
	(58)^*d*^	rs12252-C	Severe	No
	(61)	rs12252-C	Hospitalization	No
SARS-CoV-2	(69)	rs12252-C	Hospitalization	Yes
	(70)	rs12252-C	Severe	Yes
	(71)	rs12252-C	Death	Yes
	(72)	rs12252-C	Severe	Yes
	(73)	rs12252-C	Critically Ill	Yes
	(74)	rs12252-C	Hospitalization	Yes
		rs34481144-A	Hospitalization	Yes
	(75)	rs12252-C	Severe	Yes
		rs34481144-A	Severe	No
HIV-1	(76)	rs12252-C	Rapid progression	Yes
Hantaan virus	(77)	rs12252-C	Severe	Yes

^*a*^ Studies are grouped based on *IFITM3* SNP of interest and viral pathogen.

^*b*^ These studies have underpowered analysis due to low population numbers and under-representation of the *IFITM3* rs12252-C SNP.

^*c*^ These studies included a mix of both 2009 H1N1 pandemic influenza virus and seasonal influenza viruses.

^*d*^ Indicates studies that failed to find an association between the rs12252-C SNP and severe disease but do associate it with mild illness.

Supporting this, several studies on the 2009 H1N1 pandemic virus have found a strong link between *IFITM3* SNPs and severe outcomes (37, 38, 42, 51). The 2009 virus emerged from a triple reassortment of three distinct swine influenza virus strains, creating a novel virus for which the human population was immunologically naive (78–80). Everitt et al. found that patients with the rs12252-C SNP were significantly overrepresented among those hospitalized with severe 2009 H1N1 infections (38). Another study reported from China, where the SNP is common, found that nearly 70% of severe influenza cases were individuals who were homozygous for the C allele (37). This supports the notion that IFITM3 is especially important in emergent infections where there is an absence of pre-existing immunity.

Further supporting our argument, studies of other emergent viruses, such as the zoonotic H7N9 virus that spread in China in 2013, have similarly linked the rs12252-C SNP to increased inflammation and mortality (64). Another study looking at patients with either H7N9 or 2009 H1N1 infections observed that those carrying the rs12252-C SNP faced a higher risk of death, emphasizing the critical role of IFITM3 in controlling severe outcomes of emergent infections (51).

In addition, Allen et al. provided evidence from three human cohorts showing that *IFITM3* SNPs are associated with more severe disease in influenza virus infections (42). In the FLU09 cohort, carriers of the rs34481144-A allele exhibited more severe symptoms following the 2009 H1N1 infection. Examination of a Genentech challenge study cohort showed that participants with the SNP experienced higher viral titers when experimentally infected with an H3N2 virus for which all participants were serologically naive. Finally, a study of critically ill children also linked infection severity to the *IFITM3* SNP. While data are not available as to whether these children were completely naive to the influenza virus strains they were infected with, the fact that these children experienced severe disease while lacking predisposing conditions suggests that their adaptive immunity was insufficient to prevent critical illness. This reflects the heightened dependence on innate immune defenses like IFITM3 in contexts where adaptive immunity is either inadequate or absent. Collectively, these cohorts illustrate how *IFITM3* SNPs are associated with severe outcomes in scenarios where innate immunity is the primary early defense against infection (42).

However, not all studies looking at the pandemic 2009 virus reported a significant association with severe disease. One study, focused on children infected with influenza virus between 2008 and 2016, including confirmed cases of the 2009 pandemic influenza virus, reported that the rs12252-C allele was not associated with severe disease (41). This study includes seasonal and pandemic virus infections, making it difficult to determine whether the lack of association depends on pre-existing immunity (41). Two other studies looking at Spanish or Portuguese populations infected with the pandemic 2009 influenza virus determined there was no association between rs12252-C and severe infection, but the authors of both reports noted that their sample sizes were underpowered to conclusively determine a lack of association (61, 62).

Studies of seasonal influenza infections have typically failed to find a link between *IFITM3* SNPs and severe illness, potentially due to the compensatory role of adaptive immunity. One study included three groups of patients infected by seasonal strains of influenza virus and found no association between the rs12252-C SNP and severe outcomes (60). A large portion of these patients were reported to have received vaccination in the prior 6 months, reinforcing the idea that pre-existing immunity may compensate for innate immune deficiencies. Two other studies failed to find an association between the SNP and severe illness from seasonal H1N1 or H3N2 infections, but did find an association with mild infections, further supporting the dominant role of adaptive immunity in these cases (58, 59).

Importantly, *IFITM3* SNPs have been consistently linked to more severe outcomes in several other emergent infections, including Hantaan virus, HIV, and SARS-CoV-2. In the case of Hantaan virus, individuals with even one copy of the rs12252-C allele were at risk for more severe hemorrhagic fever (77). Additionally, patients carrying the rs12252-C SNP showed faster HIV progression and higher viral loads (76). The recent emergence of SARS-CoV-2 contributed to numerous studies investigating the association of *IFITM3* SNPs and COVID-19 illness (69–75). One of the first studies to look at *IFITM3* SNPs in the context of SARS-CoV-2 infection found an association between severe COVID-19 and the rs12252-C SNP (70). Other groups later confirmed this association, linking the rs12252-C SNP with a heightened risk of hospitalization, severe illness, and death due to SARS-CoV-2 infection (69, 71–75). Another study also linked the rs34481144-A SNP with an increased risk of hospitalization due to COVID-19 (74). Collectively, these findings reinforce the critical role of IFITM3 in emergent viral infections and further suggest that the compensatory role of adaptive immunity may explain the inconsistent associations observed in studies of seasonal influenza.

## ADAPTIVE IMMUNITY IN IFITM3 DEFICIENCY

While IFITM3 has been best studied as an innate immune effector protein, recent data indicate that it may have additional roles in critical signaling pathways in adaptive immune cells. Data from individuals with the rs12252-C SNP showed reduced antibody responses following vaccination against influenza virus on average, though most individuals showed antibody titers within the normal range seen in control subjects (81). Further investigation with IFITM3-deficient mice observed similar modest reductions in antibody responses following vaccination against influenza virus that were associated with decreases in activated B cells (81). IFITM3 has indeed been implicated in promoting PIP3K signaling downstream of the B-cell receptor to drive antibody affinity maturation (82). Other studies have shown that IFITM3 may also contribute to the balancing of CD4^+^ T-cell subsets (3, 83, 84). It is important to note that while these studies may indicate partially impaired responses, there is not a total absence of adaptive immunity. Unpublished work from our laboratory confirms a modest decrease in antibody titers in vaccinated IFITM3 KO animals. However, we have observed that infected KO animals are immune to subsequent re-challenge, indicating that their adaptive immune responses are functionally sufficient to compensate for the loss of IFITM3.

## PUBLIC HEALTH IMPLICATIONS

Given that most failed associations between *IFITM3* SNPs and severe influenza virus infections are from studies focused on seasonal influenza viruses where pre-existing immunity plays a dominant role in viral restriction, it is likely the most critical role of IFITM3 is in defense against emergent viruses where adaptive immunity is absent. Recent work from our group shows that IFITM3 deficiency not only increases the severity of influenza virus infections, but it also lowers the infectious dose needed to establish a productive infection with zoonotic viruses (44). Furthermore, we found that virus passaging through IFITM3-deficient hosts accelerates interspecies influenza virus adaptation (44). We have also demonstrated that IFITM3 deficiency can similarly accelerate SARS-CoV-2 adaptation in mice while still preserving variant-specific traits of the different SARS-CoV-2 isolates (85). These findings overall indicate that IFITM3 deficiencies create a vulnerability for the interspecies transmission and adaptation of zoonotic viruses, highlighting a broader role for IFITM3 in pandemic prevention efforts.

Testing of IFITM3 status in the human population could bolster pandemic prevention efforts by allowing vulnerable individuals to receive vaccine coverage targeting a wider array of influenza viruses beyond the standard seasonal strains, possibly including H5 and H7 subtypes that have commonly spilled over into humans, or to exercise enhanced precautions, such as masking, when interacting with animal reservoirs of infection or infected individuals. Furthermore, the development of IFITM3-based antiviral therapeutics could aid those with IFITM3 deficiencies and offer broad benefits to the wider population.
